# Highly-Sensitive, Label-Free Detection of Microorganisms and Viruses via Interferometric Reflectance Imaging Sensor

**DOI:** 10.3390/mi14020281

**Published:** 2023-01-21

**Authors:** Monireh Bakhshpour-Yucel, Sinem Diken Gür, Elif Seymour, Mete Aslan, Nese Lortlar Ünlü, M. Selim Ünlü

**Affiliations:** 1 Department of Electrical Engineering, Photonics Center, Boston University, Boston, MA 02215, USA; 2 Department of Chemistry, Faculty of Science and Art, Bursa Uludag University, Bursa 16059, Turkey; 3 Department of Biotechnology, Hacettepe University, Ankara 06800, Turkey; 4 Lunenfeld-Tanenbaum Research Institute, Mount Sinai Hospital, Toronto, ON M5G 1X5, Canada; 5 Department of Biomedical Engineering, Photonics Center, Boston University, Boston, MA 02215, USA

**Keywords:** pathogenic microorganisms, viruses, sensitive detection, interferometric reflectance imaging sensor, biomass measurement, nanoparticle detection

## Abstract

Pathogenic microorganisms and viruses can easily transfer from one host to another and cause disease in humans. The determination of these pathogens in a time- and cost-effective way is an extreme challenge for researchers. Rapid and label-free detection of pathogenic microorganisms and viruses is critical in ensuring rapid and appropriate treatment. Sensor technologies have shown considerable advancements in viral diagnostics, demonstrating their great potential for being fast and sensitive detection platforms. In this review, we present a summary of the use of an interferometric reflectance imaging sensor (IRIS) for the detection of microorganisms. We highlight low magnification modality of IRIS as an ensemble biomolecular mass measurement technique and high magnification modality for the digital detection of individual nanoparticles and viruses. We discuss the two different modalities of IRIS and their applications in the sensitive detection of microorganisms and viruses.

## 1. Introduction

Microorganisms such as fungi, bacteria, protozoa, and viruses can be the cause of diseases. Some microorganisms lead to diseases that might have serious health outcomes in humans and can threaten public health. They can spread by a variety of means such as air, exposure to body fluids, contaminated water and foods. Widespread transmission causes pandemics, overwhelms healthcare systems, and leads to economic losses. Therefore, rapid, direct, real-time, and label-free detection of pathogenic microorganisms is critical in ensuring rapid and accurate therapy and containing the outbreaks. In addition, the current techniques utilized in clinical laboratories have drawbacks such as long processing times and need for sophisticated instrumentation and trained personnel. The traditional methods based on the culturing are time-consuming due to the long incubation times from 24 h up to 7 days. In addition, biochemical tests are needed for identification of the isolated microorganism [1,2,3]. On the other hand, immunoassay-based tests such as enzyme linked immunosorbent assay (ELISA) and enzyme immunoassay (EIS) are fast and have capacity to quantify the target microorganism and virus particles. However, the insufficient sensitivity and specificity of these assays limit their widespread use [4]. One of the most commonly used methods for the clinical microorganism detection is the polymerase chain reaction (PCR) which depends on the amplification and quantification of the short nucleic acid sequences that exist in the target genome. Although PCR is more sensitive and less time-consuming than virus culture, the main challenges of this technique, such as the requirement for sample processing, knowledge of the sequence data of the target microorganism, costly materials, and the need for professional laboratory staff, have led researchers to develop new methods for sensitive, fast, and simple pathogen detection [5,6].

Bacterial infections have been a prevalent mortality threat in developing countries for many years due to emerging antibiotic resistance. The determination of microorganisms has been a focus of biotechnology and medical research. Especially in recent years, the inadequacies of current diagnostic methods have emerged. Together with epidemics and COVID-19 pandemic caused by viruses, the importance of early diagnosis in preventing transmission was revealed [7]. Within this context, the new biosensor devices that reduced the assay time were designed to precisely detect microorganisms and viruses at low concentrations even in complex body fluids without sample preparation steps. Highly sensitive, multiplexed, high throughput, and quantitative detection of microorganisms and viruses will be the main focus of developing new techniques [8,9].

Among different biosensor systems, surface plasmon resonance (SPR) technology using optical devices for sensing has attracted a lot of attention as it provides real-time detection without the need for any labeling. Several bacteria and viruses have been detected using this system in the literature [10,11,12,13,14]. However, the high cost of the consumables needed for SPR, e.g., gold substrates, is an important issue that restrict their widespread clinical use [6]. On the other hand, IRIS technology has shown significant advancements in microfluidic integration and allows multiplexed detection in a single cartridge. An entire IRIS cartridge costs <$5 owing to its Si-based substrate, while SPR consumables are over $200 [15]. The IRIS system has been utilized for label-free, real-time, and multiplexed quantification of molecular binding events.

The aim of this review is to focus on optical sensor systems that employ interferometric sensing with no labeling. The monitoring of molecular binding without any secondary reactants would significantly simplify the procedures of detection [9].

IRIS was developed for highly-sensitive, label-free, dynamic detection of molecular binding events in real-time and shown to have excellent sensitivity and reproducibility [16,17,18]. Recently, due to considerable advancements in IRIS technology, single-particle IRIS (SP-IRIS) was developed to visualize individual viruses for sensitive virus diagnostics applications [19,20,21]

Here, we summarize the working principles of IRIS device and developed methods for efficient preparation of the chip surface to detect low concentrations of target. Low-magnification IRIS for ensemble biomolecular mass measurements and high-magnification IRIS for digital detection of individual nanoparticles and viruses are the two different modalities. In this study, we aim to discuss the two different modalities and their applications for the detection of pathogen microorganisms, their metabolites and viruses. The IRIS system provides a multiplexed platform to allow for low-cost, rapid, and simple detection of real-time binding events without the need for complicated sample preparation.

## 2. Interferometric Reflectance Imaging Sensor

IRIS is a reflectance-based sensor which facilitates the monitoring of biomass accumulation on top of a layered substrate. The device was developed by Ünlü et al. and its working principle has been thoroughly described in the literature [16]. Briefly, a silicon chip with a thermally grown silicon dioxide (110 nm SiO_2_) is illuminated with four different colors (457, 518, 595, and 632 nm) and the change in the reflected light intensity is recorded as the biological particles bind on the sensor surface [22]. This reflected light is imaged on a CMOS (complementary metal-oxide semiconductor) camera for real-time monitoring of binding events, and the thickness of the oxide layer is precisely selected to enhance the change in the recorded intensity to accurately detect the binding events as the biomass aggregates on the sensor [23].

One of the significant advancements that distinguishes IRIS from other sensor platforms is the utilizing low-cost Si-based substrates as a multiplexed sensing cartridge. Thanks to the well-established semiconductor manufacturing industry, Si-based cartridges of IRIS platform can be produced in scalable amounts and the cost of each microfluidic cartridge is less than $5.

The efficacy of label-free, microarray-based assays depends on surface chemistry that influences the nature and reactivity of the bio-recognition elements needed to be immobilized on the chip surface. To reach a maximum binding, the immobilized probes need to be easily accessible by the targets hence they are distributed at a specific distance for enough space between them.

The surface chemistry utilized for the modification of substrate surface should meet the requirements such as accessible functional groups for probe attachment, reducing non-specific binding, stability against environmental changes, and also ideally be robust, low cost, and easily prepared. Approaches to developing surface chemistry play a fundamental role in obtaining the most efficient capture surface by immobilizing receptors onto a surface since the functionality of immobilized molecules are influenced mostly by the used immobilization process.

In the IRIS platform, prior to the spotting of the chip surface with specific ligands, the oxide surface of the silicon chip is functionalized with a different chemical agent such as epoxy silane (3-glycidoxipropyl) trimethoxysilane, MCP (co-poly-DMA-MAPS-NAS), Lucidant Polymers, LLC, USA, that provide a functional covalent link [24,25].

The IRIS instrument has evolved into its present form over the last years by optimizing some parts off the shelf and custom parts with more functional variants. Especially the expensive and bulky tunable laser source used for the illumination of the microfluidic cartridges has been replaced with narrow-band LEDs to obtain smaller-sized, low cost and easy-to-use instruments that require lower power.

The LEDs also eliminate the background artifacts caused by the coherent speckle pattern of the laser. The optical setup of IRIS carries a four-color LED source with different wavelengths (457, 518, 595 and 632 nm), an integrating sphere to obtain a uniform illumination, a condenser lens, 50:50 beam splitter, an objective, a tube lens, and a camera.

Images of four colors need to be recorded for each experiment to calibrate the reflectance signal to biomass value conversion, then the flow experiment is carried out with a single color optimized for the highest contrast. The proper LED color must be chosen according to the thickness of the SiO_2_ layer to obtain the largest reflectance signal recorded with the accumulation of biomass.

For instance, blue color (457 nm) is used with 110 nm thick oxide to obtain an accurate and more efficient image. A 2X/0.06 NA objective (Nikon) is typically used in the low magnification modality of IRIS to capture the reflected image from a large, 5 mm × 7 mm field of view (FOV) that allows multiplexed and high throughput detection.

Since IRIS provides a label-free and proper quantitation of the surface-bound biomolecules, it utilizes for quality control of bioassay chips. With a 1-inch-square field of view, biomolecule spots on a microarray prepared on an IRIS chip conforming to the size of a microscope slide, 25 × 75 mm, read and quantified by taking three images in less than 1 min. It is also possible to combine label-free calibration with standard fluorescence reading in a single instrument.

The low magnification modality of IRIS ensures quantitative, multiplexed and dynamic monitoring of binding of the surface-immobilized capture probes to their ligand on the sensor surface. Applications of this technique of IRIS contain definite quantification of protein or DNA adsorption, real-time monitoring of the binding process, and accurate measuring of the association/disassociation rates. These ensemble characteristics are extracted from series of images taken during the experiment. As the biomass accumulate on the sensor, the cumulative scattered light alters the light intensity with respect to reference region and differential images are calculated to quantify this accumulation. The relationship between light intensity and accumulated thickness is studied in detailed [26]. After this calculation, the differential signal is fitted with the model of choice on MATLAB. For more details of noise analysis and fitting models, we refer readers to the following references [15,27,28,29,30,31,32].

SP-IRIS has been developed by Ünlü group, Boston USA, from the previously explained process of IRIS. Here, the signal is based on the interference between the scattered field from the particle of attention and the reference field reflected off the interface of the layered substrate [27,28,29,30,31,32]. In this modality, the particle appears as a diffraction limited spot on the captured images. Thus, this enables detection of single bacteria and nanoparticles. Zaraee et al., demonstrated *E. coli* detection over a large-FOV (2.83 mm × 2.1 mm) using SP-IRIS [33]. They performed digital detection and counting of individual bacteria using custom developed MATLAB software. More details of this algorithm are discussed in [34].

## 3. Application of IRIS for the Detection of Nano-Scaled Microorganisms

**Figure 1:**
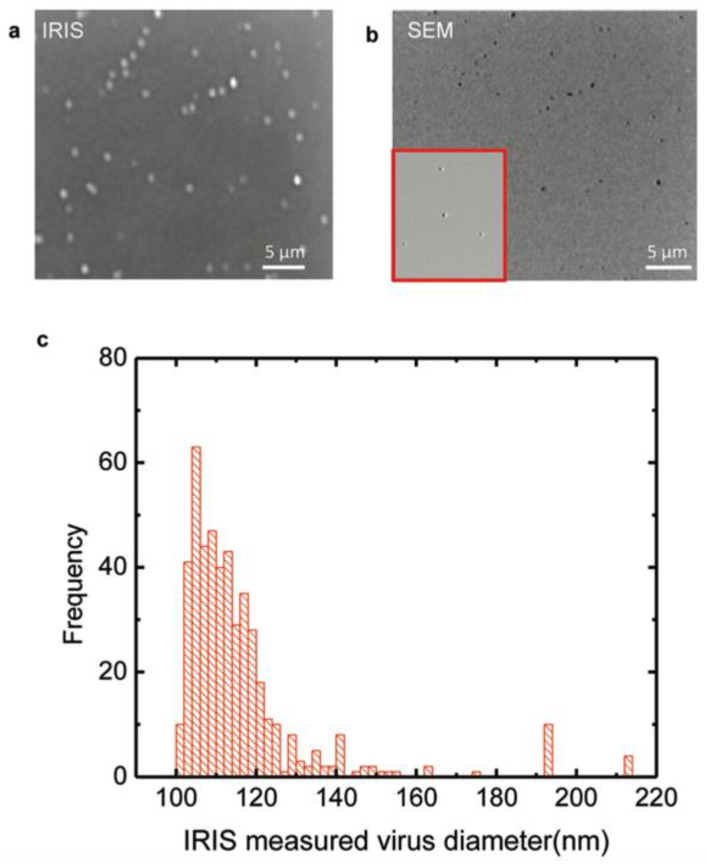
Imaging and sizing of H1N1 influenza virus. (**a**) IRIS image of captured viruses on the chip surface; (**b**) SEM photo of the same area monitored with IRIS; (**c**) size distribution of viruses measured by IRIS [20]. Reproduced with permission from G.G. Daaboul, A. Yurt, X. Zhang, G.M. Hwang, B.B. Goldberg, M.S. Ünlü, Nano Letters; Published by American Chemical Society, 2010.

The development of rapid diagnostic methods for the detection of nano-scaled pathogens that threaten human health has attracted the attentions of many researchers for decades. Some developed techniques have demonstrated promising capacity for detecting nano-sized pathogens such as viruses, however, the large sizes and high costs of these devices have prevented their widespread usage in the clinic [32,35,36]. Although, imaging most of the biomolecules is difficult with optic methods due to their small size, low contrast with environment and weak interactions with photons, the detection of influenza A has been achieved with a photonic device. However, this method was insufficient in distinguishing nanoparticles with different sizes individually from complex media depending on environmental noise. Previously mentioned limitations of optical systems for detecting and discriminating size differences of nanoparticles have been overcome with interferometric technique based on layered Si-SiO_2_ substrate which allows the interference of light reflected from the oxide surface and the oxide/silicone interface to translate into a well-defined response for each particle size and incident light of different wavelengths [20]. In this study, size discrimination and detection of individual H1N1 influenza virus were achieved with IRIS. In addition, in order to confirm the results of IRIS, scanning electron microscope (SEM) image of the virus captured area was recorded and appeared to be similar to IRIS image (Figure 1). In agreement with the literature, the mean virus size with IRIS was determined as 116–117 nm [37].

Lopez and co-workers also utilized IRIS to determine whole viruses and viral protein components of vesicular stomatitis virus (VSV) with a label-free method [19]. In this study, Si-SiO_2_ chip surface of IRIS were functionalized with copoly (DMA-NAS-MAPS) to provide efficient immobilization of viral antigen specific antibodies by covalent attachment owing to the reactive succinimide component of copolymer. After coating, mouse IgG, anti-Vaccinia (polyclonal for Lister strain) and anti-VSV-G (monoclonal for 8 G5 and 1 E9) as antibody probes were spotted on the chip surface in an array format. When the chip surfaces were incubated with wild type (wt)VSV, the detection of virions were achieved with 3.5 × 10^5^ plaque forming units/mL (PFU/mL) limit of detection, depending on the thickness change of layered substrate with the accumulated biomass. They also aimed to detect internal and external viral proteins of VSV as an alternative method for pathogen detection. With this aim, monoclonal antibodies for nucleocapsid (N) and matrix (M) protein of wtVSV were used as probes. Specific detection of virion proteins was achieved depending on the optical height change up to 5 nm on the probes of M and N proteins. Moreover, in order to confirm the results of label-free IRIS method, antibody captured chip was incubated with fluorescent labeled wtVSV sample. Although some non-specific interactions were observed, most of the fluorescent accumulation was seen at the expected probes area.

**Figure 2:**
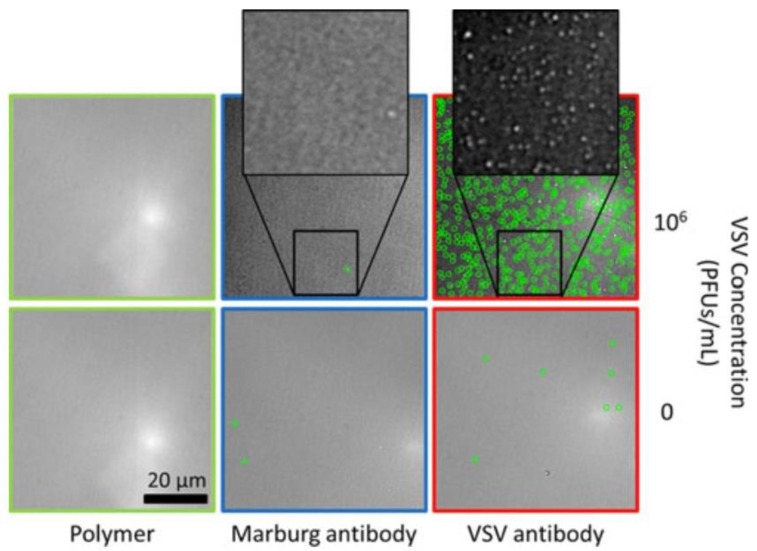
Determining the specificity of the method for VSV. VSV detection (green circles) was seen for concentration 0 and 10^6^ PFU/mL [31]. Reproduced with permission from A.P. Reddington, J.T. Trueb, D.S. Freedman, A. Tuysuzoglu, G.G. Daaboul, C.A. Lopez, W.C. Karl, J.H. Connor, H. Fawcett, M.S. Ünlü, IEEE Transactions on Biomedical Engineering; Published by IEEE, 2013.

The most important issue in clinical diagnostic tests is that the target molecule, which can be found in small amounts in the complex patient samples, can be detected sensitively and specifically despite the large quantity of intrinsic biomolecules existed in untreated body fluids. Non-specific bindings occurred depending on the intrinsic biomolecules reduce the confidence of test. The success of SP-IRIS method in detecting specific bindings is based on the use of the size discrimination algorithm, which increases the detection limit [38]. SP-IRIS was utilized to detect biosafety level 2 pathogen wtVSV from spiked fetal bovine serum (FBS) with wtVSV [31]. To evaluate the specificity of the method, chip surface was decorated with both anti-VSV (8 G5 clone) and as a negative control, monoclonal antibody specific to Marburg virus. After incubation of spotted chip surface with 5 × 10^5^ PFU/mL of wtVSV spiked FBS, particles with 100–140 nm diameters were observed at the anti-VSV spotted area thanks to the CMOS camera (GS2-GE-50S5M-C, Point Grey Research, Inc., Richmond, BC, Canada) of SP-IRIS (Figure 2). However, it is known that the bullet-shaped VSV (80 nm × 160 nm) is observed in the form of a spherical particle with a diameter of 110 nm when viewed under non-polarized light [21].

**Figure 3:**
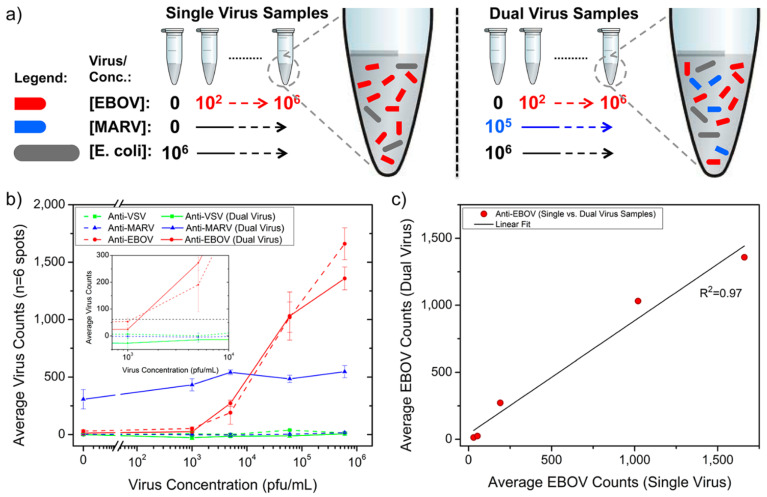
Dual detection of pseudotyped EBOV and MARV from fetal bovine serum contaminated with *E. coli* K12 at the concentration of 10^6^ CFU/mL. Scheme of (**a**) preparation of single and dual virus spiked samples; (**b**) the results obtained for single virus detection and dual detection at different virus concentrations; (**c**) the response obtained with anti-Ebola spots for FBS and bacteria spiked FBS samples was similar, as indicated by the linear regression fitting the scatter plot of one sample type versus the other [39]. Reproduced with permission from G.G. Daaboul, C.A. Lopez, J. Chinnala, B.B. Goldberg, J.H. Connor, M.S. Ünlü, ACS Nano; Published by American Chemical Society, 2014.

Daaboul and coworkers took the SP-IRIS technique one step further and demonstrated the success of SP-IRIS in detecting multiple viruses from highly bacteria-contaminated complex samples (serum or whole blood) [38]. The thickness of Si-SiO_2_ substrate is optimized for imaging viruses and nanoparticles with sizes in the range of 60–200 nm. The layered sensor surface was designed in a microarray format with antibodies specifically recognized the glycoproteins of VSV, Ebola (EBOV) and Marburg virus (MARV). Ebola and Marburg glycoproteins were obtained by inserting related gene region with pAK vector into the VSV genome where the VSV glycoprotein-encoded sequence was deducted. The results were seen to be correlated when the responses obtained for samples containing individual pseudo type viruses (EBOV and MARV) were compared with those obtained for the sample in which the two viruses were co-existing. The limit of detection was recorded as 5 × 10^3^ PFU/mL for EBOV and MARV in both the mixed and single virus existed whole blood samples spiked with 10^6^ colony forming units/mL (CFU/mL) of *E. coli* K12 to increase the sample complexity (Figure 3).

**Figure 4:**
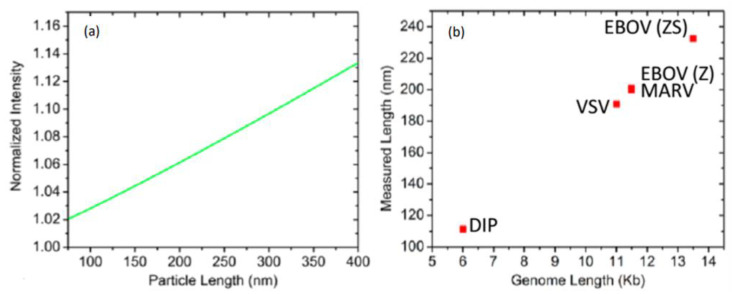
Discrimination of viruses with genome length. (**a**) The sizing curve that was composed with the responses obtained for varying lengths of a cylindrical particle with constant width, was used to calculate the dimension of virus particles based on VSV captured on the SP-IRIS chip surface. (**b**) The virus lengths measured with SP-IRIS for wtVSV, DIP, MARV, EBOV (Z), EBOV (ZS) [39]. Reproduced with permission from G.G. Daaboul, C.A. Lopez, J. Chinnala, B.B. Goldberg, J.H. Connor, M.S. Ünlü, ACS Nano; Published by American Chemical Society, 2014.

Moreover, variant VSVs (wtVSV, defective interfering particles of VSV (DIP), Ebola pseudotyped VSV- Ebola Zaire (EBOV-Z), Ebola Zaire/Sudan (EBOV-ZS), Marburg pseudotyped VSV (MARV)) with different genome lengths and correspondingly different sizes which were obtained the insertion of gene regions of different lengths, were incubated with the chip. As a result, it was dedicated that SP-IRIS was an efficient tool not only to achieve discrimination between each virion and background particles existed in the blood sample but also for the same virus with a size difference of 20 nm between them (Figure 4). In this study, they combined probe capturing in a microarray design, target recognition based on affinity and digital sensing to achieve rapid and sensitive virus detection by sizing and counting of individual virus. They also indicated that SP-IRIS was the first method which combines label-free detection in a microarray format with digital visualization of single bio-nanoparticle (virion).

**Figure 5:**
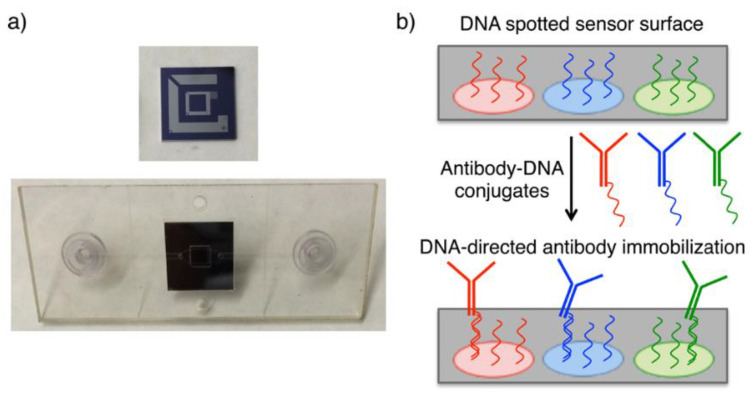
(**a**) Photos of IRIS chip and microfluidic cartridge. (**b**) Schematization of ssDNA spotted sensor area and antibody immobilization on the chip surface through DNA hybridization [44]. Reproduced with permission from E. Seymour, G.G. Daaboul, X. Zhang, S.M. Scherr, N.L. Ünlü, J.H. Connor, M.S. Ünlü, Analytical Chemistry; Published by American Chemical Society, 2015.

Protein microarray-based detection approaches have gained attention for biomedical applications such as pathogen or biomarker detection, drug development and screening [40,41]. However, some limitations may be encountered in the immobilization of proteins to the array surface depending on surface chemistry. While capturing antibodies to the surface, multiple covalent bindings that can be occurred between antibody and the surface, are causing activity lose by blocking antigen binding sides of the antibody [41,42]. Additionally, steric hindrance between antibody-surface and antibody-antibody may affect activity. These challenges have led researchers to investigate alternative protein binding methods in order to enhance sensitivity of assays [43]. Seymour and coworkers utilized a DNA associated probe immobilization technique that combines the advantages of DNA microarray with diagnostic benefit of proteins by using DNA conjugated antibodies [44]. Thus, DNA conjugated antibodies were immobilized onto the chip with their complementary ssDNA probes instead of forming antibody array on the sensor chip surface (Figure 5).

**Figure 6:**
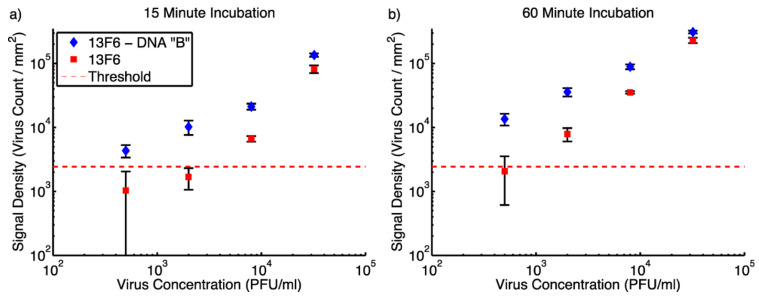
Comparison of the detection efficiency of directly immobilized VSV-pseudotyped EBOV antibodies with DNA-conjugated types. The measurement of signals obtained by captured viruses with SP-IRIS at different virus concentrations after (**a**) 15 min incubation (**b**) 60 min incubation [44]. Reproduced with permission from E. Seymour, G.G. Daaboul, X. Zhang, S.M. Scherr, N.L. Ünlü, J.H. Connor, M.S. Ünlü, Analytical Chemistry; Published by American Chemical Society, 2015.

Owing to the advantages of this method, such as increasing the capacity of antigen binding [45], providing homogeneous distribution of the probes [46], and enhancing the reproducibility of the assay [47], they have improved the existed SP-IRIS technology by creating a versatile and robust sensing platform. DNAs conjugated to antibodies acting as spacer arms, allowing antibodies to rise on the polymeric surface. Due to the reduced steric hindrance depending on the elevation of antibodies, capturing efficiency of antigenic substances was enhanced. Firstly, they optimized the length of DNA probes immobilized on the polymeric surface by utilizing spectral self-interference fluorescents microscope (SSFM) in order to develop high-throughput virus sensing platform. To compare the efficacy of DNA conjugated antibody binding with direct antibody binding, genetically modified VSV (containing the gene encoding the Ebola glycoprotein) was used as the model virus. DNA sequences named as A and B were used to conjugate monoclonal 8G5 (anti-wtVSV) and monoclonal 13F6 (anti-EBOV pseudotyped VSV), respectively. On the other hand, the same antibodies were spotted on the polymeric surface without DNA linkers. As a result of the study, it was seen that after 15 min incubation LOD was determined as 500 PFU/mL for DNA conjugated 13F6 whereas 8000 PFU/mL for directly immobilized antibody. Thanks to the increased capture efficiency, sensitive and specific virus detection has been acquired even in a short incubation time (Figure 6).

**Figure 7:**
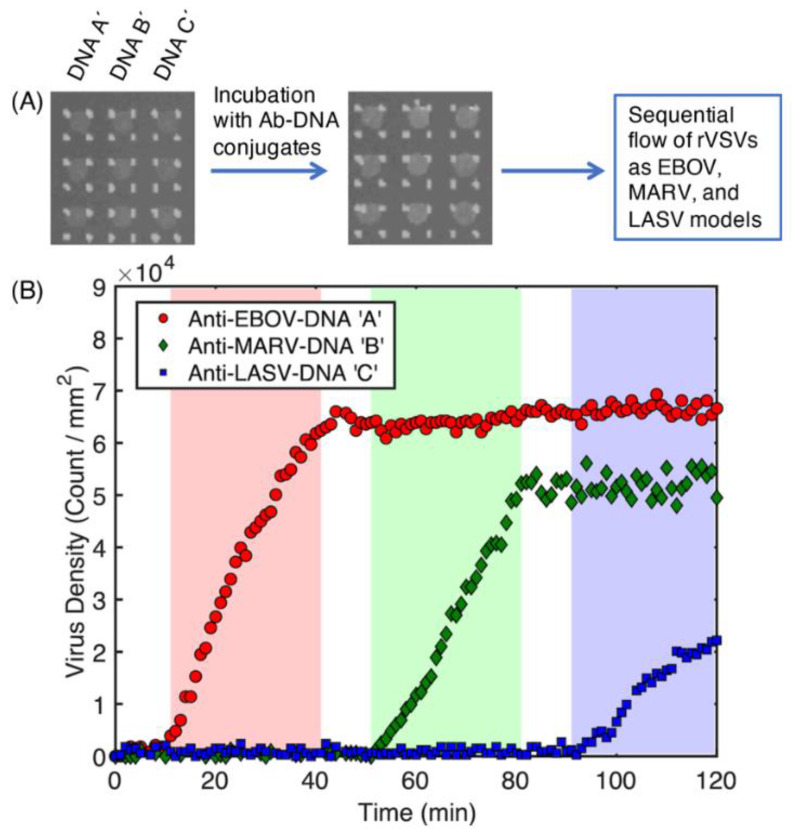
Multiplexed and real-time virus detection with SP-IRIS on a chip designed with DNA conjugated antibodies. (**A**) The SP-IRIS chip surface spotted with three different DNA sequences and incubation with DNA conjugated antibodies (anti-EBOV, anti-MARV, anti-LASV). (**B**) Virus density of each DNA conjugated antibody spots following sample flow [48]. Reproduced with permission from E. Seymour, N.L. Ünlü, E.P. Carter, J.H. Connor, M.S. Ünlü, ACS Sensor; Published by American Chemical Society, 2021.

After the optimization of DNA-based constitution of chip surface, this method was used for multiplex virus detection [48]. VSV-pseudotyped Ebola, Marburg and Lassa virus specific antibodies (anti-EBOV, anti-MARV, anti-LASV) conjugated with ssDNAs were immobilized on the chip surface decorated with complementary ssDNAs. After antibody immobilization, virus samples were sequentially passed through the microfluidic cartridge with the following order; VSV-EBOV, VSV-MARV and VSV-LASV. When each virus was flowed through the cartridge for 30 min, signal increase was seen in its specific antibody spots. An increase in virus densities was measured with SP-IRIS during experiment (Figure 7). DNA conjugated antibody immobilization was proved as an effective method for designing multiplexed antibody array. Moreover, antibody sets can be changed to detect different targets during the need with this programmable DNA-based chip surface.

**Figure 8:**
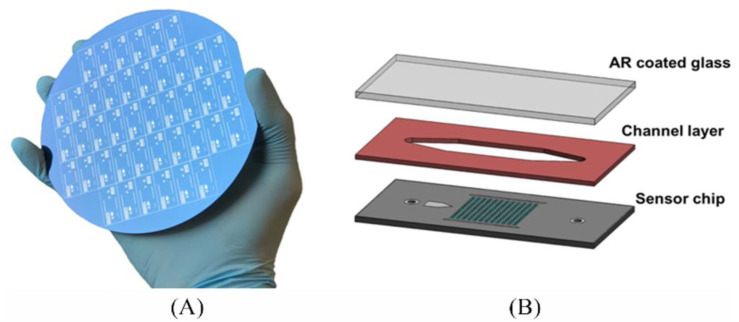
Schematic representation of new generation. (**A**) IRIS chip; (**B**) Si-based IRIS cartridge [49]. Reproduced with permission from A.Y. Ozkumur, F.E. Kanik, J.T. Trueb, C. Yurdakul, M.S. Ünlü, IEEE Journal of Selected Topics in Quantum Electronics; Published by IEEE, 2019.

Diagnostic approaches that are utilized to create point of care assay should be not only sensitive and rapid but also easy to use and inexpensive. In a recent study of SP-IRIS, low cost (less than $10) polymer/plastic glass cartridges incorporated with paper-based fluid carrying system have been developed instead of previously used cartridges, which cost $40–100 [49]. The microfluidic cartridge is including a Si-SiO_2_ sensor chip with 100 nm thickness that has input and output holes, a cover glass (0.7 mm thick) that coated with anti-reflection on the chip to form flow chamber and a silicone spacer (0.5 mm thick) between the glass and the chip (Figure 8). Improvements have also been made to the instrumentation by replacing high-cost camera and translation stages in order to obtain a low-cost (less than $10,000) and portable prototype without compromising the gains of the method such as individual imaging and multiplex detection. Dynamic detection of Ebola virus-like particle (VLP) was demonstrated by using anti EBOV-VLP in this study.

*E. coli* which is a Gram-negative bacteria, is one of the most common contaminants of water and food [50]. Additionally, *E. coli* is the causative agent of many diseases that can be fatal if not treated. Thus, early diagnosis of pathogen is an important in order to prevent the spread of pathogen and increase the survival rate [51].

**Figure 9:**
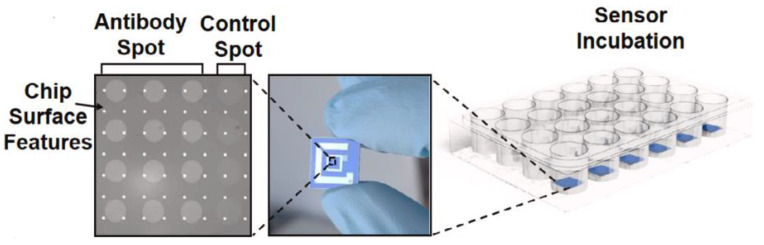
Schematization of IRIS chip surface decorated in an antibody array format with anti-*E.coli* and bovine serum albumin as a control. Experiment stage: incubation of spotted IRIS chips with 1 mL of serial *E. coli* concentrations in the 24-well plate [33]. Reproduced with permission from N. Zaraee, A.M. Bhuiya, E.S. Gong, M.T. Geib, N.L. Ünlü, A.Y. Ozkumur, J.R. Dupuis, M.S. Ünlü, Biosensors and Bioelectronics; Published by Elsevier, 2020.

In a study of Zaraee and coworkers, whole cell pathogenic bacteria detection and visualization were achieved with SP-IRIS for the first time [33]. Anti-*E. coli* spotted IRIS chips were incubated with serial dilutions of *E. coli* culture in a concentration range of 10–10^6^ CFU/mL in the 24-well plate for 2 h (Figure 9). They reached 2.2 CFU/mL limit of detection and also visualized individually captured rod-shaped *E. coli*. Moreover, tap water spiked with different concentrations of *E. coli* was used as a real sample. Increased average particle counting on per mm^2^ of antibody spotted area was observed with the increased concentrations of *E. coli*. The specificity of the sensor was evaluated with using Gram-positive *Staphylococcus aureus*, Gram-negative *Pseudomonas aeruginosa* and *Klebsiella pneumonia*. Due to the insignificant cross reactivity seen for non-target bacteria, it was mentioned that SP-IRIS platform shows great specificity to the target pathogen when realized multiplex detection.

## 4. Discussion

IRIS technology is a promising device for whole-cell microbial detection, as well as for the detection of small molecular weight microbial metabolites. Fumonisin B1, a mycotoxin with high toxicity, is produced by *Fusarium* spp. Fumonisin B1, which threatens animal and human health, can be found as a contaminant in cereal grains and products [52]. The food industry is faced with the critical problem of accurate detection of the presence of toxin in food products during the quality control process. Chiodi and coworkers designed a chip for IRIS to determine low molecular weight Fumonisin B1 [53]. Twenty antibodies tested for this toxin were spotted on the chip surface with different concentrations. When detecting mass accumulation of small sized biomolecules, to obtain enhanced sensitivity level more of the chip surface should be used or temporal resolution need to be reduced. The signal noise must be lower than the binding signal measured at the CMOS camera for determining binding event with the biomass accumulation on the IRIS chip surface. The noise level of 3 ng/mm^2^ that recorded for single pixel without any improvement, prevents the detection of larger molecules such as antibodies. With the temporal and spatial averaging applied in this study, sensitivity of 1.0 pg/mm^2^ was obtained by increasing the signal to noise ratio. Moreover, the results obtained with IRIS were compared with SPR which is a gold standard method for label-free small molecule detection. The sensitivity of the results was shown to agree with the data obtained by SPR. However, the traditional SPR method has challenges such as the incapability to perform multiplex assays and the utility of high-cost metal chips (more than $200). Although the more complex SPR imaging (SPRi) can be used for multiplex detection, the limit of detection of SPRi remains low for the identification of small molecules [54].

Additionally, IRIS utilized to detect the lethal factor of anthrax toxin that responsible for disease [15]. The toxin is produced by *Bacillus anthracis* which can be used as a powerful agent for bioterrorism. Bacterial toxins that pass into the bloodstream can cause death despite antibiotic treatment [55]. Determination of specific antibodies that can neutralize the toxin is important in developing treatment [56]. Marns and coworkers accomplished scanning of most specific antibody ligands for anthrax lethal factor (ALF) by the antibody array-based IRIS method and showed that the results conform to SPR [15]. Multiple ligands were screened to compare capability of binding ALF in the same experiment setup owing to the multiplex assay capacity of IRIS. Considerable data that used for developing treatment and diagnosis can be reached by the obtained data of binding kinetic and complementary binding behavior from multiplex tests. The k_on_ and k_of_ values based on the dynamic binding of the LF antigen to two different single chain antibodies were found to be similar to the results obtained with SPR [57]. IRIS proved its success to monitor binding events between antigen and antibody simultaneously.

## 5. Conclusions

The first step in an effective treatment of infection diseases is the correct and rapid diagnosis of the causative agent. Culturing, immunoassays, molecular methods such as polymerase chain reaction are the main techniques used for pathogen detection in clinical laboratories. However, the different challenges seen in each of these methods such as being time consuming, requirement for expensive supplies and professional staff, need for additional sample preparation step, lack of sensitivity or specificity have led researchers to new discoveries in recent years. In this review, firstly advantages and disadvantages of the conventional methods used in the clinic to detect microorganisms and viruses were summarized. Then, the working principles of a newly developed method IRIS were clarified and finally successful applications of this method based on visualization of a single particle in terms of size and shape were summarized.

## Data Availability

Not applicable.
